# Systemic delivery of anti-sense oligonucleotide targeting α-synuclein for treatment in a mouse model of multiple system atrophy

**DOI:** 10.3389/fnagi.2026.1807721

**Published:** 2026-04-29

**Authors:** Brian Spencer, Bao Quach, Sahar Salehi, Robert A. Rissman

**Affiliations:** Department of Physiology and Neuroscience, Epstein Family Alzheimer's Therapeutic Research Institute, Keck School of Medicine of the University of Southern California, San Diego, CA, United States

**Keywords:** alpha synuclein, anti-sense oligonucleotides, multiple system atrophy, systemic therapy, transgenic mouse

## Abstract

**Introduction:**

Multiple System Atrophy (MSA) is a rare, sporadic, age-related synucleinopathy characterized by Parkinson-like motor symptoms and ataxia. There is no therapy for MSA other than symptomatic treatment. MSA is characterized pathologically by glial cytoplasmic inclusions (GCI) of α-synuclein (αSyn) occurring in oligodendrocytes leading to loss of myelination in the brain.

**Methods:**

We recently utilized a peptide-mediated delivery method to systemically transport an anti-sense oligonucleotide (ASO) targeted to αSyn in a mouse model of MSA. We hypothesized that systemic delivery of αSyn ASO by peptide mediated delivery to a mouse model of MSA would reduce the αSyn accumulation in oligodendrocytes and reduce the overt pathology associated with MSA.

**Results:**

Following monthly treatments of the αSyn ASO, we found increased myelination in the corpus callosum, cerebellum and brainstem. We also observed increased numbers of oligodendrocytes and reduced gliosis; however, we did not detect changes in overall αSyn in the areas of the brain we examined. Upon further analysis, we determined the peptide-mediated delivery of αSyn ASO was not taken up by oligodendrocytes.

**Conclusion:**

Thus, we have successfully alleviated some of the pathology associated with MSA in a mouse model; however, without direct delivery to oligodendrocytes, other approaches may need to supplement this therapy.

## 1 Introduction

Multiple system atrophy (MSA) is a rare, sporadic, age-related synucleinopathy characterized by accumulation of α-synuclein (αSyn) in the form of glial cytoplasmic inclusions (GCI) in the oligodendrocytes of the central nervous system, leading to myelin loss, neuronal loss and neuronal inflammation (Spillantini et al., 1998; Wakabayashi et al., 1998; Woerman et al., 2018). MSA presents as two unique neurodegenerative diseases characterized by loss of neurons in the striatonigral regions leading to Parkinsonian-like motor symptoms like bradykinesia and postural instability (MSA-P) or cerebellar ataxia (MSA-C).

MSA-C is characterized by αSyn accumulation initially within the pontine cerebellar projections and cerebellar white matter, which is accompanied by pathological phosphorylation at Ser129 (P-Syn) (Nishie et al., 2004). To date it is controversial how oligodendrocytes accumulate pathological αSyn; however, clear evidence exists that oligodendrocyte dysfunction is characterized by accumulation of cytoplasmic inclusions containing αSyn and P-Syn which leads to myelin loss and ultimately to loss of neurons as well as gliosis (reviewed in Ahmed et al., 2012). Currently there is no disease modifying therapy available for MSA, and even symptomatic treatment is limited, particularly for MSA-C.

Currently available transgenic mouse models of MSA over-express αSyn under oligodendrocyte specific promoters such as the proteolipid promoter (Kahle et al., 2002) and the cyclic nucleotide phosphodiesterase promoter (Yazawa et al., 2005). We characterized a transgenic mouse model of MSA-C (MBP29) developed by over-expressing human αSyn with the oligodendrocyte specific myelin basic protein promoter (MBP) resulting in accumulation of αSyn in GCI of oligodendrocytes (Shults et al., 2005). This model expresses and accumulates αSyn in the cortex, basal ganglia, corpus callosum and the cerebellum (Shults et al., 2005; Ubhi et al., 2010; Valera et al., 2017). Meszaros characterized the cerebellar accumulation of αSyn in the MBP29 mouse model of MSA and concluded that the model can best be described as a model of MSA-C based on myelin deficits in the cerebellar white matter, loss of Purkinje neurons in the cerebellum and motor deficits including decreased walking speed and gait instability (Meszaros et al., 2021). All this is associated with αSyn accumulation in the oligodendrocytes suggesting the MBP29 mouse is a model of MSA-C.

Synuclein degrading enzymes such as neurosin have proven effective at reducing the accumulation of αSyn in the MBP29 mouse model of MSA-C. Addition of a 38 amino acid LDL-R binding domain from Apolipoprotein B (ApoB^38^) facilitated delivery across the blood-brain barrier following systemic administration (Spencer et al., 2015). Although the ApoB^38^ was not very effective at delivering neurosin to oligonucleotides, reduction in oligodendroglial αSyn was observed. In an attempt to reduce the expression of αSyn protein and thus prevent the accumulation of αSyn, we attempted to deliver anti-sense oligonucleotides (ASO) targeting αSyn mRNA.

ASO therapy is an effective means for modifying expression of genes. Delivery of a short 20–30 nucleotide sequence anti-sense to the specific gene transcript in affected cells can reduce protein expression by either steric hinderance or activation of RNase and RNA degradation (reviewed in Lauffer et al., 2024). ASOs and related splice switching oligonucleotides delivered for neurodegenerative diseases are either in clinical trials or in some cases approved therapeutics (Lozupone et al., 2023; Cantara et al., 2024; Li and Bedlack, 2024; Shafie et al., 2024). However, ASO therapies for neurodegenerative disorders require intra-thecal administration because the ASO does not cross the blood–brain barrier (BBB). We have developed a peptide mediated transport approach for the delivery ASOs to the brain following systemic administration.

In the process of developing a peptide for delivery of ASO, we were able to shorten the transport peptide from 38 amino acids to the 11 amino acids (ApoB^11^) necessary and sufficient for binding to the LDL-R and transport across the blood-brain barrier (Spencer et al., 2019; Leitao et al., 2023b; Ahammad et al., 2025a,b). We have shown the ability of the ApoB^11^ to transport an ASO sequence targeted to αSyn for a systemic therapy for synucleinopathies that has proven effective in synucleinopathy models of Parkinson's disease and Dementia with Lewy Bodies (Spencer et al., 2019; Leitao et al., 2023a). In this study, we examined whether this new ApoB^11^ peptide would better target therapeutic delivery to oligonucleotides and/ or whether an ASO targeted to αSyn would reduce the expression of the transgenic αSyn and affect myelination in a mouse model of MSA.

To determine if systemic peptide mediated delivery of an αSyn ASO could be an effective therapy for the αSyn accumulation in oligodendrocytes of MSA, we delivered the ApoB^11^-αSyn ASO by intra-peritoneal injection monthly for 2 months to female MBP29 mice that model MSA-C. We hypothesized that delivery of αSyn ASO to the mouse model of MSA-C would increase myelination and oligodendrocytes and reduce neuroinflammation.

## 2 Materials and methods

### 2.1 Synuclein transgenic mice

For this project, female mice expressing the human αSyn under the control of the myelin basic protein promoter (MBP29) were used (Shults et al., 2005). We used the MBP29 line as these animals express a high level of αSyn in oligodendrocytes in the corpus callosum, cortex, cerebellum, and brainstem along with associated loss of myelin and neuroinflammation beginning at 2–3 months of age (Shults et al., 2005; Ettle et al., 2016; Hoffmann et al., 2019; Meszaros et al., 2021).

Female, transgenic and non-transgenic littermates were used for this experiment as previous characterization of the MBP29 mouse model of MSA-C was performed only on female mice (Ettle et al., 2016). Mice received two intra-peritoneal injections of the peptide-ASO complex at a dosage of 2 mg/kg (volume 10 mL/kg) 4 weeks apart beginning at 3 months of age (Supplementary Figure S1). Treatment dose and frequency were determined from previous successful ASO delivery and pharmacokinetic analysis (Spencer et al., 2019; Leitao et al., 2023a; Ahammad et al., 2025a,b). Experiment length was limited to 2 months as the average life span of the MBP29 mouse is 5–6 months.

Treatment involved a synthetic peptide, ApoB^11^, conjugated with a 2′-*O*-methyl (2′-OMe) antisense oligonucleotide (ASO) we previously showed targeted human and mouse αSyn (Spencer et al., 2019; Leitao et al., 2023a). ApoB^11^ peptide (NH_2_-RLTRKRGLKLAGGGGGRRRRRRRRR) was synthesized to 95% purity (GenScript, Piscataway, NJ) and resuspended in nuclease-free water. αSyn ASO (2′MOE 5′GAC TTT CAA AGG CCA AGG A) and a scrambled control (Scr) ASO (2′MOE 5′GGG CAT ACT GAG CTA ACA A) (Integrated DNA Technology, San Diego, CA) were synthesized, purified, and resuspended in RNase-free TE buffer. The ApoB^11^ peptide and the respective ASO were incubated in PBS to form peptide-ASO complexes at a ratio of 10:1 for 30 min at room temperature prior to injection as previously described (Leitao et al., 2023a). At least four animals per group were treated and all animals were sacrificed 4 weeks after receiving the final treatment.

All experimental procedures involving animals were conducted at UCSD and were approved by the Institutional Animal Care and Use Committee of UCSD, following the NIH Guide for the Care and Use of Laboratory Animals (protocol #S02221). Efforts were made to minimize animal suffering and to reduce the number of animals used.

### 2.2 Anatomical studies

After the completion of treatments, mice were euthanized in accordance with NIH guidelines followed by brain extraction. Right hemi-brains were immersion-fixed in 4% phosphate-buffered formaldehyde for 5 days at 4 °C and subsequently sectioned sagittally at 40 μm with a vibratome. The left hemi-brains were snap-frozen in liquid nitrogen for biochemical analysis.

For neuropathological examination, serial brain sections between 1.25 and 1.75 mm lateral to Bregma were immunolabeled overnight at 4 °C with antibodies specific to total αSyn (BD Transduction Laboratories, San Diego, CA, Cat#610787, RRID:AB_398108), phosphorylated αSyn (Ser129) (Abclonal, Woburn, MA, Cat#AP0450), RRID:AB_2771549, Olig2 (Novus Biologicals, Centennial, CO), Cat#NBP1-28667, RRID:AB_1914109), myelin basic protein (MBP, Abcam, Waltham, MA, Cat# ab218011), RRID:AB_2895537, GFAP (Millipore, Temecula, CA, Cat#MAB3402), RRID:AB_94844) and Iba1 (FUJIFILM Wako Pure Chemical Corporation, Richmond, VA), Cat#019-19741, RRID:AB_839504. The sections were then incubated with biotinylated secondary antibodies (Vector Laboratories, Newark, CA) and visualized using an avidin-biotin complex (ABC Elite; Vector Laboratories) followed by diaminobenzidine (DAB) staining. Slides were scanned using the NanoZoomer S60 Digital Slide Scanner (Hammamatsu, 20X). Regions of interest were isolated using NDP.View2 and analyzed with CellProfiler or ImageJ. For each group of animals, at least four brains were imaged and one image from each subregion was analyzed.

### 2.3 Double immunolabeling and fluorescence co-labeling

To determine the co-localization of the systemically delivered αSyn ASO and individual cells or αSyn protein, we constructed a biotinylated αSyn ASO (Integrated DNA Technology). This αSyn ASO was the same sequence as delivered for the rest of the experiments with the addition of 5′-terminal biotin. Five-month-old female MBP-αSyn tg and non-tg mice (*N* = 3 each) received a single intra-peritoneal injection of ApoB^11^-αSyn biotin-ASO at 4 mg/kg. Three days after injection, mice were sacrificed and perfused with ice cold PBS to clear blood from vessels and then 4% paraformaldehyde to fix the brain. Brains were sectioned on a vibratome at 40 μm thickness and sections between 1.25 and 1.75 mm from Bregma were incubated overnight with tyramide signal amplification direct (red; ThermoFisher, Carlsbad, CA) to label the biotinylated ASO and antibodies for Olig2 (Novus Biologicals), αSyn (BD Transduction Laboratories), NeuN (Millipore, Cat# MAB377, RRID:AB_2298772), or GFAP (Millipore) overnight at 4 °C. Sections were then incubated with species appropriate secondary antibodies labeled with FITC (green; Vector Laboratory) and mounted with ProLong Gold Antifade (Fisher Scientific, Carlsbad, CA). Sections were imaged by laser scanning confocal microscopy with a Leica Stellaris 8. Quantitation was performed with Fiji using the Squassh plugin. αSyn (Syn1) or oligodendrocyte (Olig2) positive cells were masked and the percentage of cells co-staining for the ASO was quantified. To determine oligodendrocytes that contained the ASO, the Olig2 mask was expanded by five pixels to account for the perinuclear localization of ASO.

### 2.4 Biochemical analysis

Frozen left hemi-brains from four mice per group were sub-dissected into cortex, cerebellum, and brainstem regions. These regions were homogenized [1 × nuclease free PBS, RNAse inhibitor (NEB), protease inhibitor (Mini-Complete, Roche, Carlsbad, CA)] with Bead Mill 24 (Fisher) using 1.4 mm ceramic beads (Fisher). Following homogenization, samples were removed for protein and incubated with 10 × RIPA. Protein was quantified by BCA assay (BioRad, Irvine, CA).

Immunoblotting was performed with 25 μg protein per lane loaded in a 12% Bis-Tris SDS-PAGE gel (Criterion TGX, BioRad) and transferred onto PVDF membranes using semi-dry Trans-Blot Turbo Transfer System (BioRad). The membranes were probed with antibodies against total αSyn, phosphorylated αSyn, myelin basic protein, and GAPDH (Signaling Technology, Cat#2118, RRID:AB_561053 followed by species appropriate HRP conjugated antibodies (BioRad). Proteins were visualized using enhanced chemiluminescence (SuperSignal West Pico PLUS, ThermoScientific), and quantification was performed using a ChemiDoc Imaging System (BioRad). Band intensities were normalized to GAPDH levels and compared to non-transgenic controls. Blots were stripped (Restore Western blot stripping buffer, ThermoScientific) and re-probed successively in the following order: P-Syn, T-Syn, and GAPDH and with a separate blot probing MBP and GAPDH.

For analysis of αSyn with a native gel, the same protein extracts were diluted in Native Protein sample buffer (BioRad) and 25 μg of protein was loaded in a 12% Bis-Tris gel (BioRad) and then transferred to PVDF membranes. Blots were probed with the same αSyn antibody and then stripped and probed for ß-actin levels for use as a loading control. Band intensities were analyzed with ImageLab (BioRad).

RNA was extracted from the remaining homogenized sample (*N* = 4 for each group of animals) using RNAeasy kit (Qiagen) and quantified by spectrophotometry readings. For cDNA synthesis, 500 ng of total RNA was reverse transcribed using iScript gDNA Clear cDNA Synthesis kit (BioRad). Real time-PCR (RT-PCR) was performed using IDT primetime gene expression master mix (IDT) with primer/probe designed for mouse myelin basic protein (IDT, Mm.PT.58.28532164), and human αSyn (IDT, Hs.PT.58.912923). Samples were analyzed with a real time PCR system (CFX96 Real Time System, BioRad). The amount of cDNA was calculated by the comparative threshold cycle method and expressed using mouse actin (Mm.PT.39a.22214843.g; IDT) as an internal control and then this was normalized to the control group of wild type mice treated with ApoB^11^:Scr ASO.

### 2.5 Statistical analysis

All statistical analyses were conducted using GraphPad Prism v10.0. Group comparisons were made using two-way ANOVA with Tukey's test among groups. Significance was set at *p* < 0.05. Results are expressed as mean ± standard error of the mean (SEM).

## 3 Results

### 3.1 αSyn ASO prevents reduced myelination seen in MBP-αSyn tg mice

**Figure 1:**
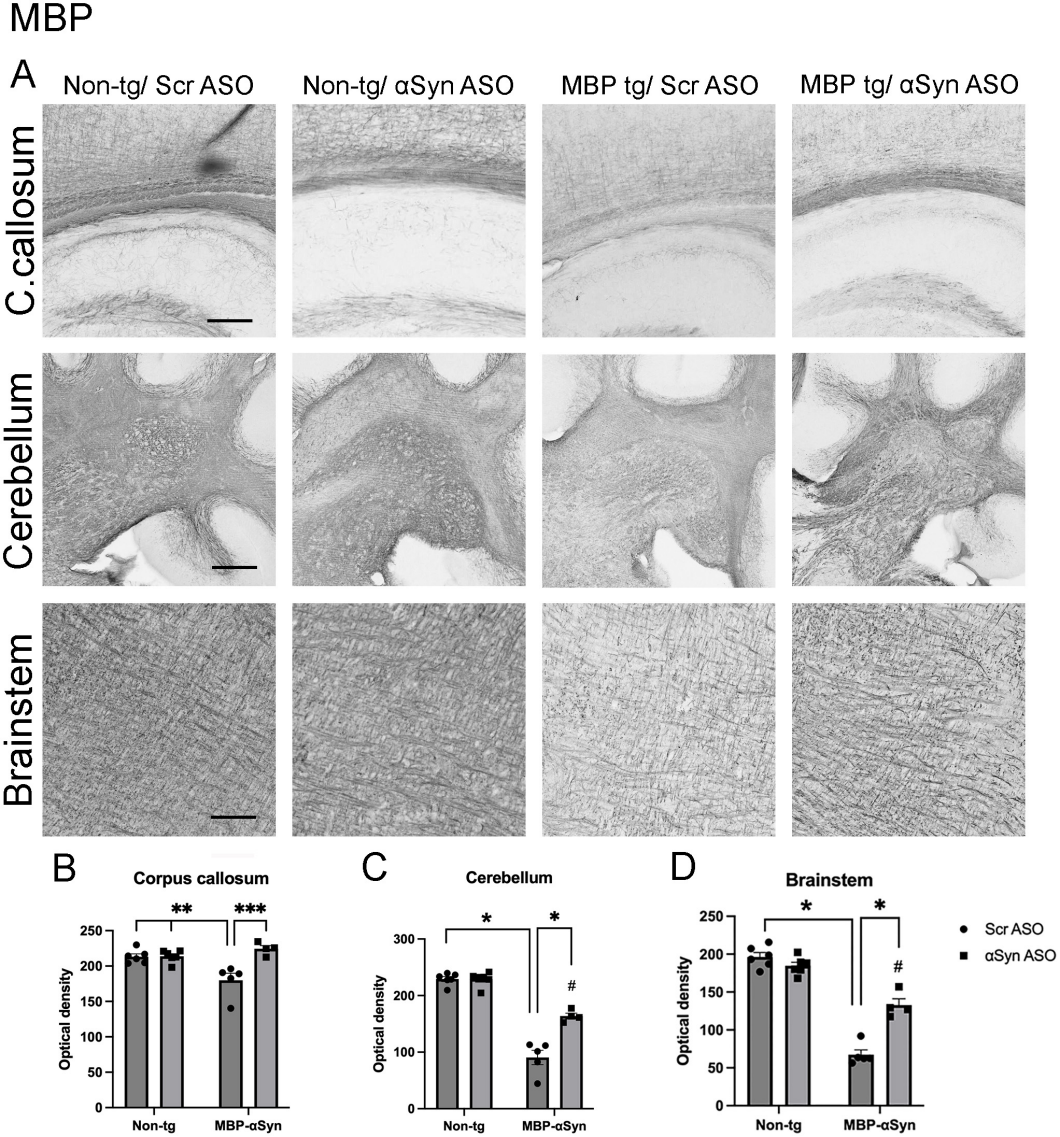
MBP staining in MBP-αSyn tg and non-tg mice treated with ApoB^11^-Scr ASO and αSyn ASO. **(A)** Representative immunohistochemistry images showing MBP staining in various brain regions, including the corpus callosum (C. callosum), cerebellum and brainstem. Scale bars = 100 μm for C. callosum and brainstem; 200 μm for cerebellum. Quantification of MBP adjusted optical density is an average of two locations in the **(B)** C. callosum, **(C)** cerebellum, and **(D)** brainstem in non-tg and MBP-αSyn-tg mice treated with Scr ASO or αSyn ASO corrected for background staining in the cortex. Data represent mean ± SEM, *p < 0.05, **p < 0.01, ***p < 0.001. #*p* < 0.05 compared to non-tg control. At least four mice per group were analyzed. Statistical analysis was conducted with two-way ANOVA (mixed model) with Tukey's test among groups.

Oligodendrocytes are responsible for myelination of neurons and MSA patients show reduced myelination associated with αSyn accumulation in oligodendrocytes (Meszaros et al., 2021). Previous characterization of the MBP-αSyn tg line 29 mice has shown reduced myelination in the corpus callous and the cerebellum (Shults et al., 2005; Spencer et al., 2015; Ettle et al., 2016; Hoffmann et al., 2019; Meszaros et al., 2021). To determine if the 11 amino acid ApoB peptide (ApoB^11^) conjugated to αSyn ASO could have a therapeutic effect on multiple system atrophy, we treated 3-month-old female MBP29-αSyn tg mice or age and sex matched non-tg littermates with either ApoB^11^-αSyn ASO or the control ApoB^11^-Scr ASO by intra-peritoneal injection twice at 4 weeks apart (Supplementary Figure S1). We stained sections from the mice with myelin basic protein (MBP) and found MBP-αSyn tg mice had reduced MBP staining in the corpus callosum, cerebellum and brainstem as previously reported indicating a loss of myelin (Figure 1A). Treatment of the MBP-αSyn tg mice with the ApoB^11^-αSyn ASO resulted in significantly increased MBP staining in the all areas examined (Figures 1B–D).

**Figure 2:**
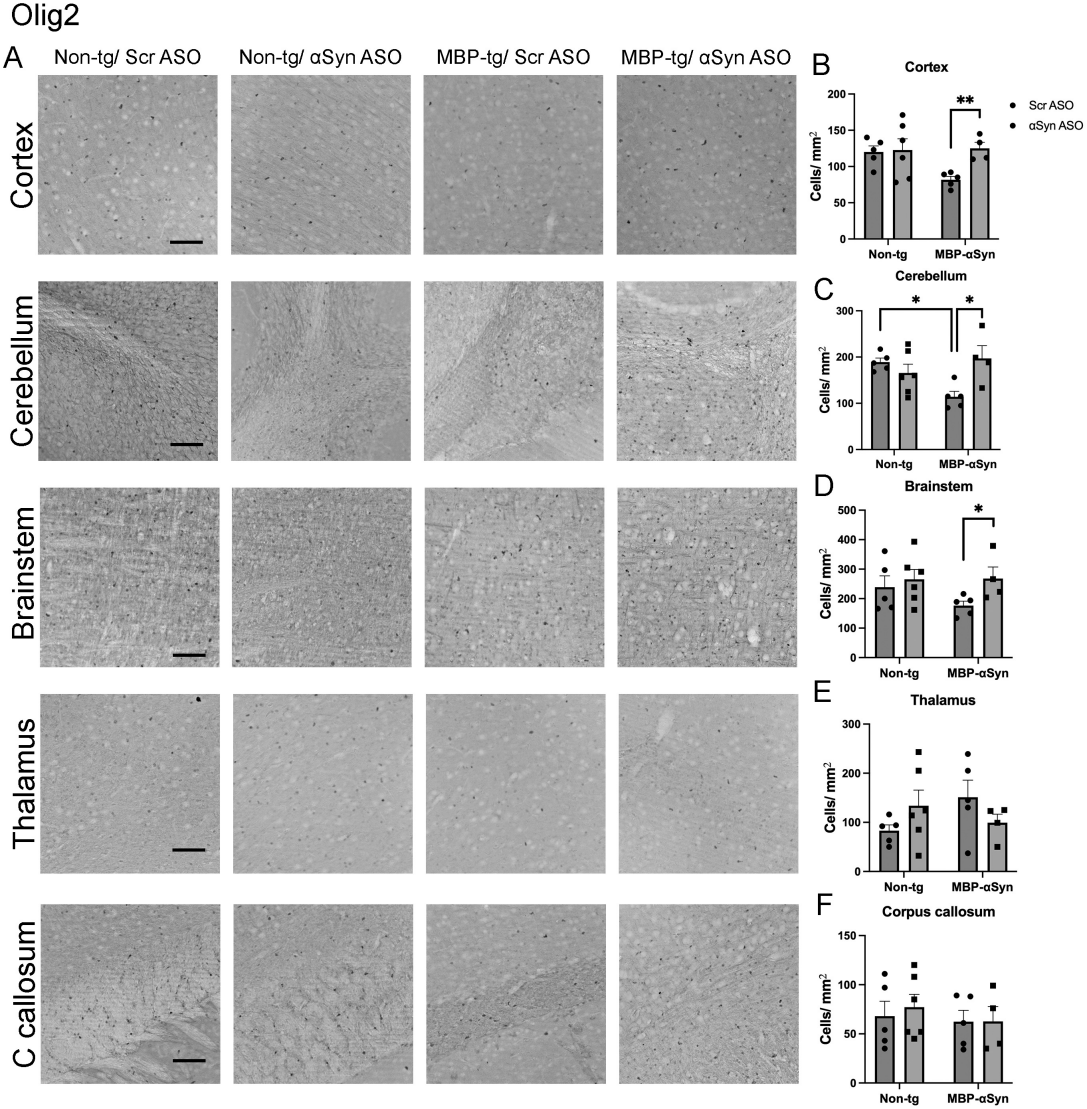
Olig2 staining in MBP-αSyn tg and non-tg mice treated with ApoB^11^-Scr ASO and αSyn ASO. **(A)** Representative immunohistochemistry images showing Olig2 staining in various brain regions, including the cortex, cerebellum and brainstem. Scale bars = 100 μm. Quantification of Olig2 positive cells/ mm^2^ in the **(B)** cortex, **(C)** cerebellum, **(D)** brainstem, **(E)** thalamus, and **(F)** C. callosum in non-tg and MBP-αSyn-tg mice treated with Scr ASO or αSyn ASO. Data represent mean ± SEM, *p < 0.05, **p < 0.01. At least four mice per group were analyzed. Statistical analysis was conducted with two-way ANOVA (mixed model) with Tukey's test among groups.

MBP-αSyn tg mice have been shown to have reduced number of oligodendrocytes in the cortex, brainstem and corpus callosum at 5 months of age despite elevated numbers at younger ages (Ettle et al., 2016; Meszaros et al., 2021). Similarly, we observed reduced oligodendrocytes in the cortex, cerebellum and brainstem as determined by immunohistochemistry with the Olig2 antibody at 5 months of age (Figures 2A–D). We did not observe a change in the number of oligodendrocytes in the corpus callosum or thalamus (Figures 2E, F). Treatment with ApoB^11^-αSyn ASO restored the number of oligodendrocytes in the cortex, cerebellum and brainstem to levels observed in non-tg mice (Figures 2B–D). Thus, systemic delivery of ApoB^11^-αSyn ASO prevented the loss of oligodendrocytes and myelination in the MBP-αSyn tg mice.

### 3.2 Treatment with αSyn ASO and accumulation of αSyn and PSyn

**Figure 3:**
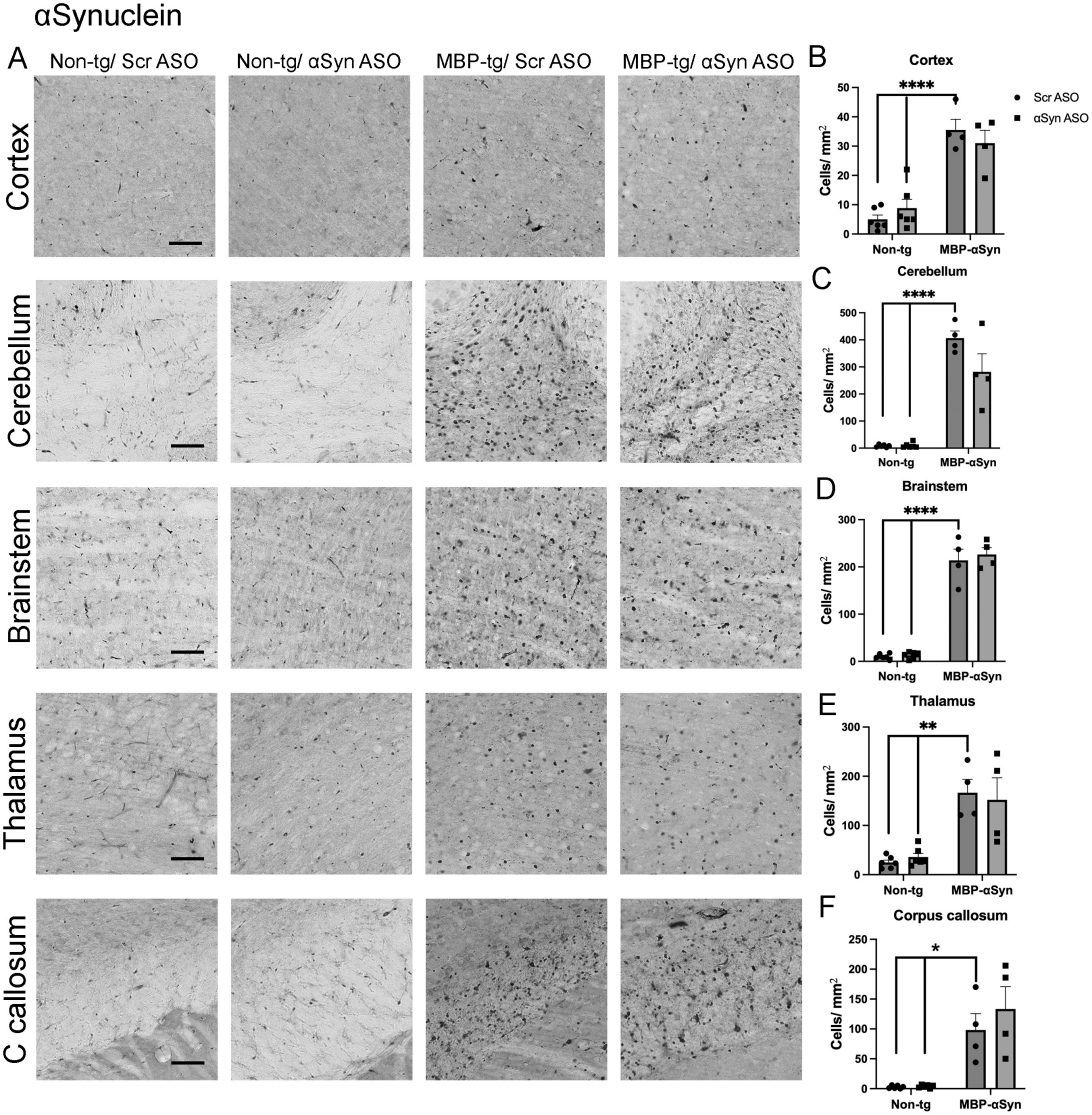
αSyn accumulation in MBP-αSyn tg (MBP-tg) and non-tg mice treated with ApoB^11^-Scr ASO and αSyn ASO. **(A)** Representative immunohistochemistry images showing αSyn staining in various brain regions, including the cortex, cerebellum and brainstem. Scale bars = 100 μm. Quantification of αSyn positive cells/mm^2^ in the **(B)** cortex, **(C)** cerebellum, **(D)** brainstem, **(E)** thalamus, and **(F)** C. callosum in non-tg and MBP-αSyn-tg mice treated with Scr ASO or αSyn ASO. Data represent mean ± SEM, *p < 0.05, **p < 0.01, ****p < 0.0001. At least four mice per group were analyzed. Statistical analysis was conducted with two-way ANOVA (mixed model) with Tukey's test among groups.

MBP29-αSyn tg mice overexpress and accumulate human αSyn in oligodendrocytes throughout the CNS but particularly in the cortex, corpus callosum, cerebellum and brainstem (Shults et al., 2005). To determine if an ASO targeted to αSyn could reduce the oligodendrocyte accumulation of αSyn, we systemically delivered ApoB^11^-αSyn ASO to transgenic and non-transgenic mice. As previously reported for MBP-αSyn tg mice, we observed αSyn accumulation in the cortex, corpus callosum and cerebellum (Figures 3A–C, E). We also examined the brainstem and thalamus where αSyn accumulated in small punctate locations (Figures 3A, D, F). Delivery of the ApoB^11^-αSyn ASO did not significantly affect the number of cells accumulating αSyn in any region examined although a relative reduction in the number of cells accumulating αSyn in the cerebellum was observed (Figure 3C).

**Figure 4:**
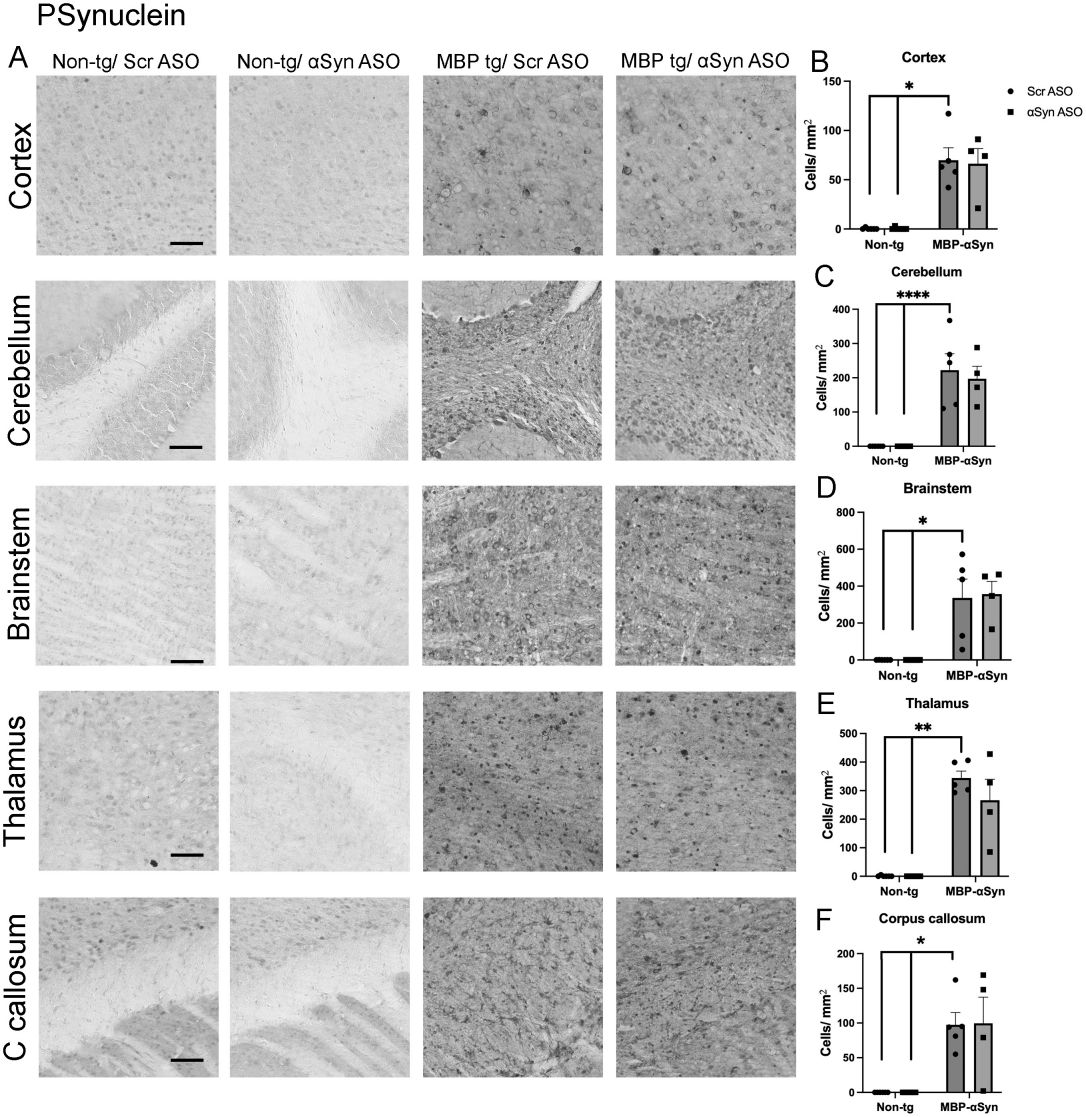
Phosphorylated αSyn (Ser129) (PSyn) accumulation in MBP-αSyn tg (MBP-tg) and non-tg mice treated with ApoB^11^-Scr ASO and αSyn ASO. **(A)** Representative immunohistochemistry images showing PSyn staining in various brain regions, including the cortex, cerebellum and brainstem. Scale bars = 100 μm. Quantification of PSyn positive cells/ mm^2^ in the **(B)** cortex, **(C)** cerebellum, **(D)** brainstem, **(E)** thalamus, and **(F)** C. callosum in wild type and MBP-αSyn-tg mice treated with Scr ASO or αSyn ASO. Data represent mean ± SEM, *p < 0.05, **p < 0.01, *p* < 0.05, ****p < 0.0001. At least four mice per group were analyzed. Statistical analysis was conducted with two-way ANOVA (mixed model) with Tukey's test among groups.

Phosphorylation of αSyn at Ser129 (PSyn) has been identified in GCI and has been linked to αSyn aggregation and neuronal loss (Nishie et al., 2004). Mouse brain sections were stained for the Ser129 PSyn. As previously reported, we observed significant accumulation of PSyn in the MBP-αSyn tg mice in the cortex, cerebellum and corpus callosum compared to non-tg mice (Figures 4A–C, E). We extended this study to examine the brainstem and thalamus where we also observed significant PSyn accumulation (Figures 4A, D, F). Treatment of the MBP-αSyn tg mice with the ApoB^11^-αSyn ASO did affect the number of cells with accumulated PSyn in any region examined.

### 3.3 αSyn ASO impact on gliosis

**Figure 5:**
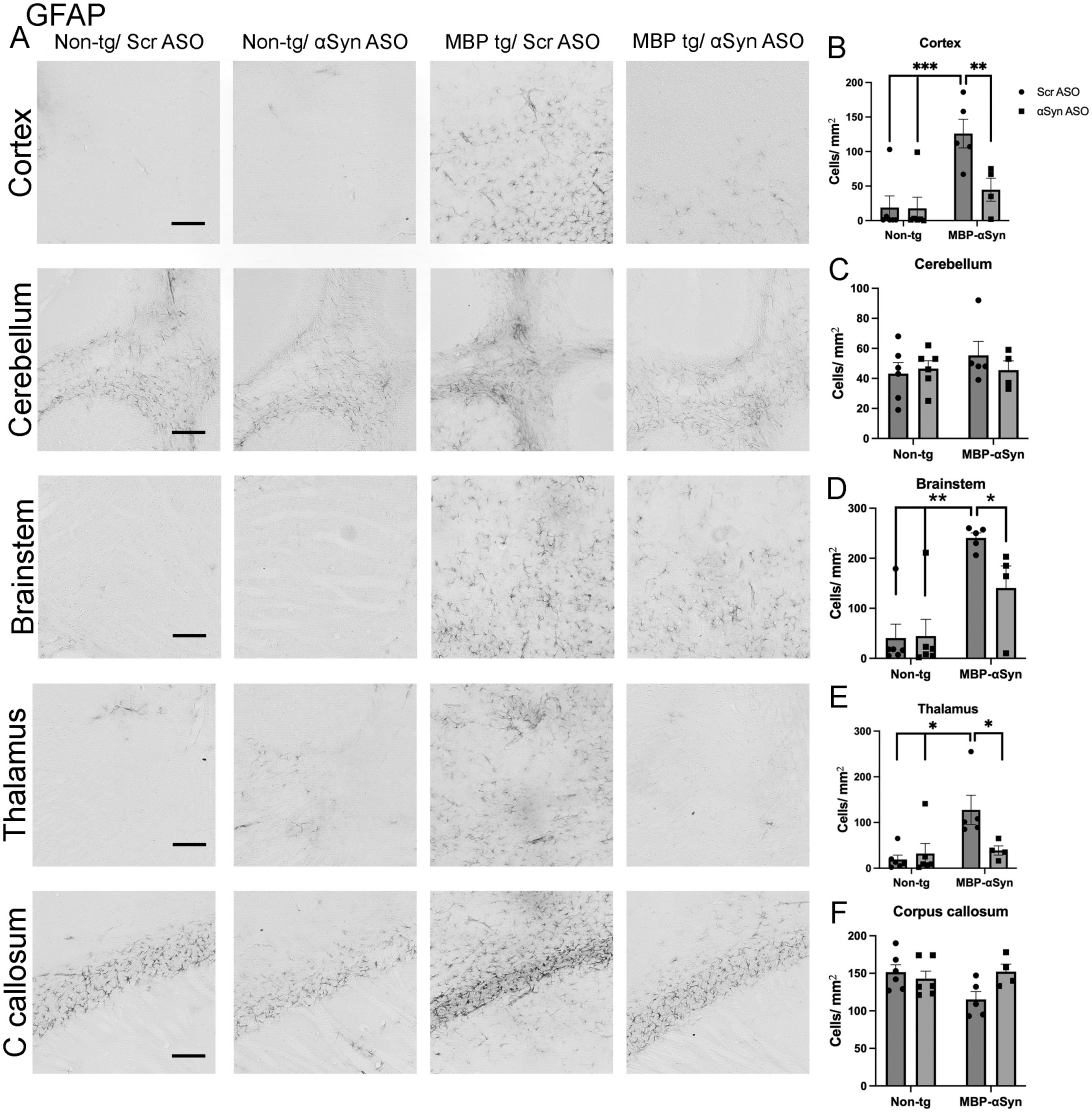
GFAP staining in MBP-αSyn tg and non-tg mice treated with ApoB^11^-Scr ASO and αSyn ASO. **(A)** Representative immunohistochemistry images showing GFAP staining in various brain regions, including the cortex, cerebellum and brainstem. Scale bars = 200 μm. Quantification of GFAP positive cells/mm^2^ in the **(B)** cortex, **(C)** cerebellum, **(D)** brainstem, **(E)** thalamus, and **(F)** C. callosum in non-tg and MBP-αSyn-tg mice treated with Scr ASO or αSyn ASO. Data represent mean ± SEM, *p < 0.05, **p < 0.01, ***p < 0.001. At least four mice per group were analyzed. Statistical analysis was conducted with two-way ANOVA (mixed model) with Tukey's test among groups.

Increased gliosis has been shown in mouse models of synucleinopathies as well as post-mortem in patients with PD and MSA (Valera et al., 2017; Hoffmann et al., 2019; Kondo et al., 2024). Indeed, the MBP-αSyn mouse model of MSA has elevated astrocytosis in the cortex and corpus callosum and increased microglia (Shults et al., 2005; Hoffmann et al., 2019; Meszaros et al., 2021). We confirmed these findings showing elevated astrocytosis in the cortex, brainstem and thalamus in MBP-αSyn tg mice (Figures 5A, B, D, F). Treatment with the ApoB^11^-αSyn ASO reduced the number of astrocytes in the cortex, brainstem and thalamus to levels observed in non-tg littermates. We did not observe increased astrocyte numbers in the cerebellum or corpus callosum (Figures 5A, C, E).

**Figure 6:**
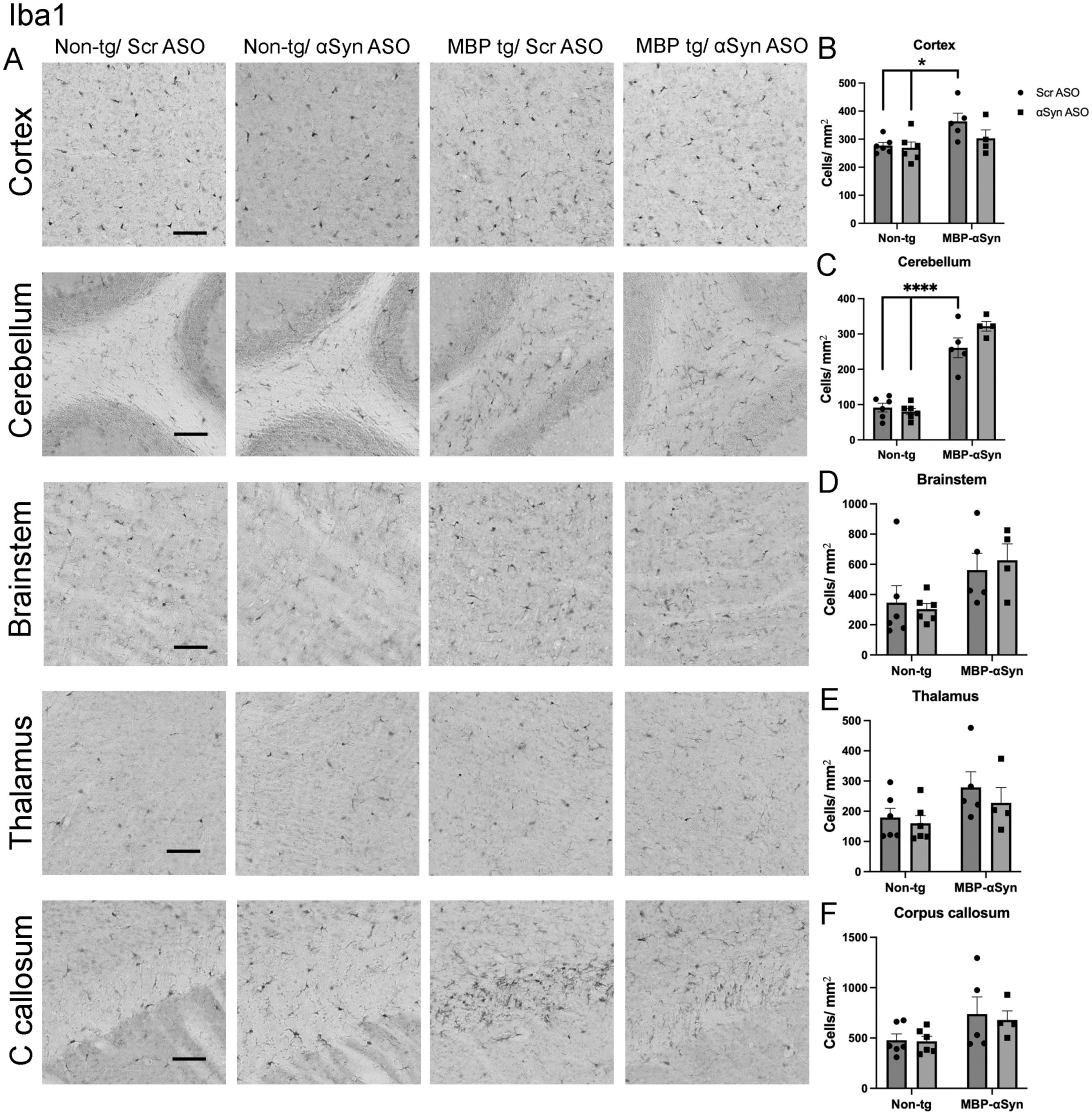
Iba1 staining in MBP-αSyn tg and non-tg mice treated with ApoB^11^-Scr ASO and αSyn ASO. **(A)** Representative immunohistochemistry images showing Iba1 staining in various brain regions, including the cortex, cerebellum and brainstem. Scale bars = 50 μm. Quantification of Iba1 positive cells/mm^2^ in the **(B)** cortex, **(C)** cerebellum, **(D)** brainstem, **(E)** thalamus, and **(F)** C. callosum in non-tg and MBP-αSyn-tg mice treated with Scr ASO or αSyn ASO. Data represent mean ± SEM, *p < 0.05, ****p < 0.0001. At least four mice per group were analyzed. Statistical analysis was conducted with two-way ANOVA (mixed model) with Tukey's test among groups.

Similarly, we observed increased number of microglia in the cortex, cerebellum, brainstem, corpus callosum and thalamus of MBP-αSyn tg mice (Figures 6A–F). In contrast to the reduction of astrocytes, we did not measure a significant change in microglial numbers following treatment with the αSyn ASO, suggesting the treatment effect was limited to astrocytes.

**Figure 7:**
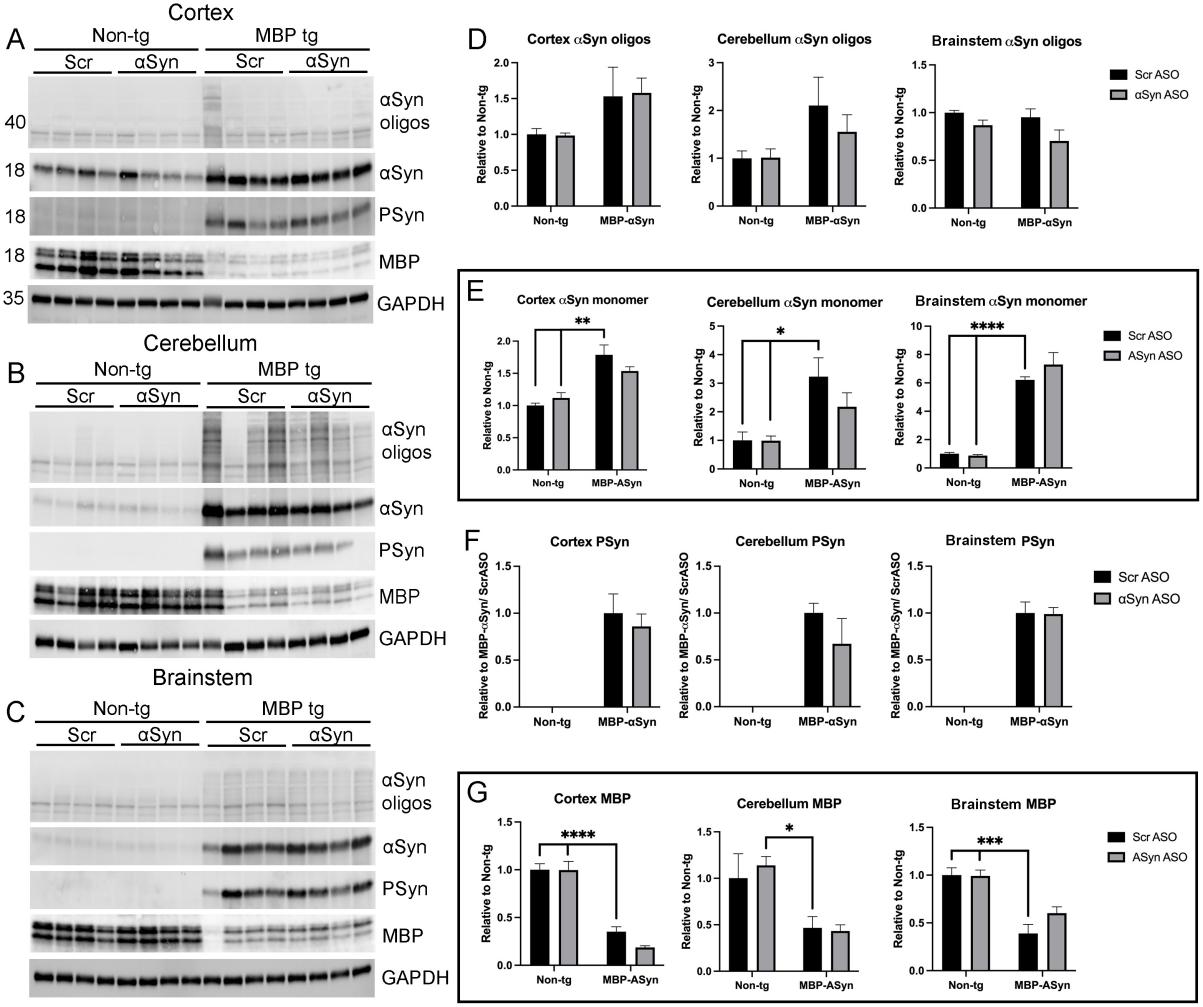
Immunoblot analysis of αSyn, PSyn, and myelin basic protein (MBP) in MBP-αSyn tg and non-tg mice treated with ApoB^11^-Scr ASO or αSyn ASO. Frozen hemi-brains were sub-dissected to isolate cortex, cerebellum and brainstem regions. Representative immunoblots from **(A)** cortex, **(B)** cerebellum, and **(C)** brainstem enriched regions analyzed with antibodies specific for αSyn monomers (18 kDa) and oligomers (>36 kDa), P-αSyn (18 kDa), MBP (14-20 kDa), and GAPDH (35 kDa). Graphs represent quantitation of immunoblots showing relative levels of **(D)** oligomeric αSyn, **(E)** monomeric αSyn, **(F)** PSyn, and **(G)** MBP protein normalized to GAPDH and then normalized to wild type treated with ApoB^11^-Scr ASO except PSyn which was normalized to MBP-αSyn tg mice treated with ApoB^11^-Scr ASO. Data represent mean ± SEM, *p < 0.05, **p < 0.01, ***p < 0.001, ****p < 0.00001. *N* = 4 mice per group. Statistical analysis was conducted with two-ANOVA (mixed model) with Tukey's test among groups.

To confirm the IHC effects observed, we homogenized brain regions enriched in the cortex, cerebellum and brainstem for immunoblot analysis (Figures 7A–C) and qPCR for gene expression (Supplementary Figure S2). Oligomeric αSyn (Figure 7D), monomeric αSyn (Figure 7E), and Ser129 phosphorylated αSyn (PSyn) were detected at elevated levels in the MBP-αSyn tg mice compared to non-tg mice as expected (Figure 7F). Similar to results from IHC, we did not measure changes in the accumulation of αSyn or PSyn in any of the brain regions analyzed following treatment with the ApoB-αSyn ASO. This was confirmed by analysis of αSyn with a native protein gel (Supplementary Figure S4). αSyn RNA levels were elevated in the MBP-αSyn tg mice compared to wild type; however, no change was detected following delivery of ApoB^11^-αSyn ASO (Supplementary Figure S2). We next assessed levels of myelin basic protein (MBP) in each of the same brain regions. Similar to results from MBP histochemistry, we measured significantly reduced levels of MBP in all three brain regions analyzed similar to previous reports (Shults et al., 2005; Meszaros et al., 2021); however, treatment with ApoB^11^-αSyn ASO did not significantly affect the levels of MBP protein with only a small increase observed in the brainstem (Figure 7G). Interestingly, RNA levels of MBP appeared to be unchanged between MBP-αSyn tg and wild type mice (Supplementary Figure S2).

### 3.4 ApoB^11^-αSyn ASO has little colocalization with oligodendrocytes or αSyn

**Figure 8:**
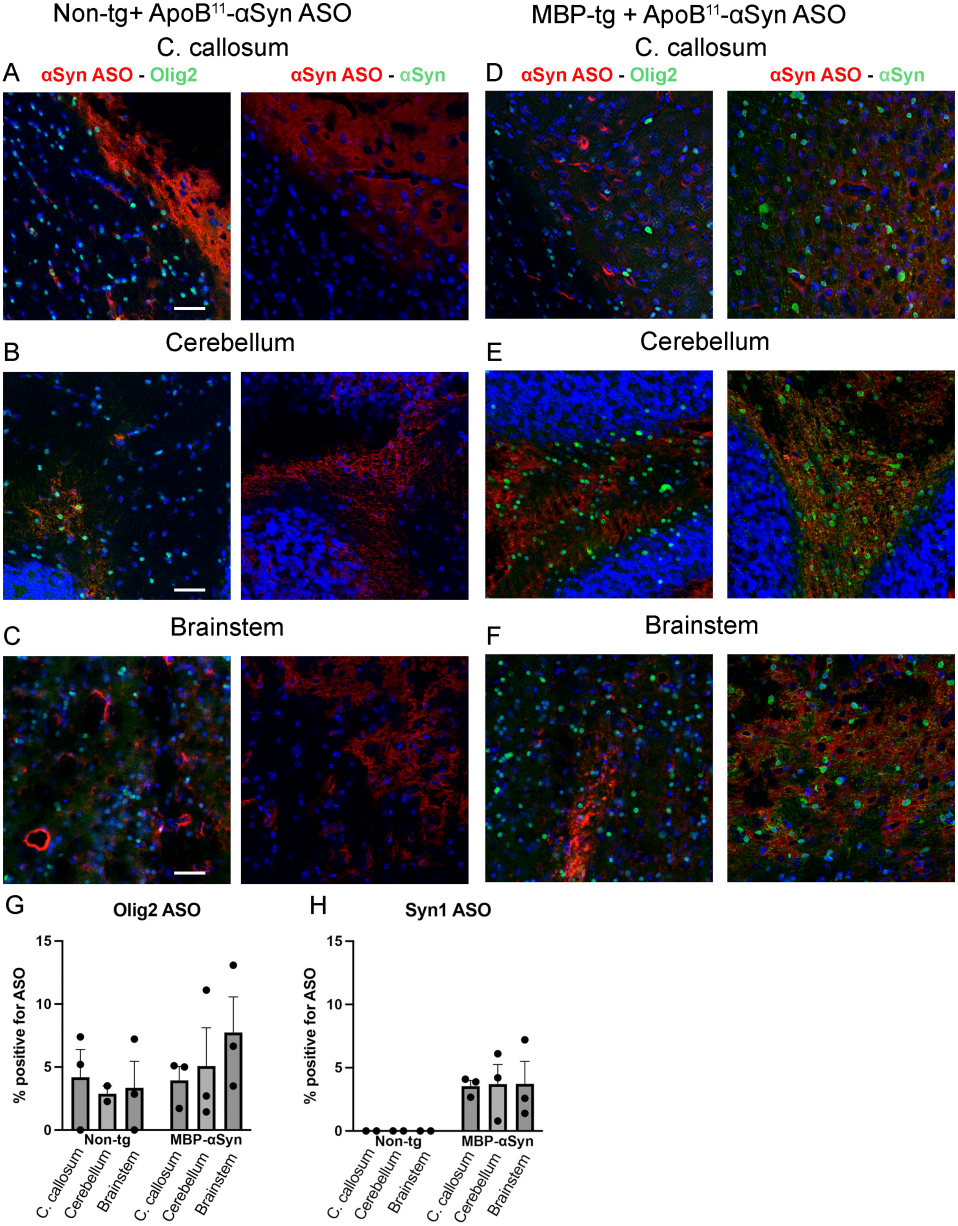
Co-localization of biotinylated αSyn ASO with oligodendrocytes (Olig2) and αSyn. Representative regions double labeled for αSyn ASO (red) and oligodendrocyte (Olig2, green) or αSyn (green) in **(A–C)** non-tg and **(D–F)** MBP-αSyn tg mice treated with ApoB^11^-αSyn ASO (biotinylated) in **(A, D)** corpus callosum, **(B, E)** cerebellum, and **(C, F)** brainstem imaged with a LSCM. Scale bars = 100 μm. *N* = 3 mice per group. Graphs represent percentage of cells co-staining for **(G)** oligodendrocytes (Olig2) and **(H)** αSyn (Syn1) and ASO. Uncropped immunoblots cortex: Original immunoblots used to generate Figure 8A. 25ug total protein from sub-dissected cortex per lane was loaded in a 15% Bis-Tris SDS-PAGE gel and transferred to nitrocellulose membrane using semi-dry transfer. A molecular weight marker (BioRad Precision Plus Protein Dual Color Standard) was loaded to visualize protein size. Blots were probed with antibodies for PSyn, αSyn, MBP, Calbindin and GAPDH followed by species appropriate secondary with horseradish peroxidase and visualized with chemiluminescence. Blots were imaged on a ChemiDoc Imaging System. Blots were stripped and reprobed successively in the following order: P-Syn, T-Syn, MBP and GAPDH, and with a separate blot: MBP, calbindin and GAPDH. Blots were imaged on a ChemiDoc Imaging System. Some residual chemiluminescence can be observed from previous antibodies used. Uncropped immunoblots cerebellum: Original immunoblots used to generate Figure 8B. 25ug total protein from sub-dissected cerebellum per lane was loaded in a 15% Bis-Tris SDS-PAGE gel and transferred to nitrocellulose membrane using semi-dry transfer. A molecular weight marker (BioRad Precision Plus Protein Dual Color Standard) was loaded to visualize protein size. Blots were probed with antibodies for PSyn, aSyn, MBP, Calbindin and GAPDH followed by species appropriate secondary with horseradish peroxidase and visualized with chemiluminescence. Blots were imaged on a ChemiDoc Imaging System. Blots were stripped and re-probed successively in the following order: P-Syn, T-Syn, MBP and GAPDH, and with a separate blot: MBP, calbindin and GAPDH. Blots were imaged on a ChemiDoc Imaging System. Some residual chemiluminescence can be observed from previous antibodies used. Uncropped immunoblots brainstem: Original immunoblots used to generate Figure 8C. 25ug total protein from sub-dissected brainstem per lane was loaded in a 15% Bis-Tris SDS-PAGE gel and transferred to nitrocellulose membrane using semi-dry transfer. A molecular weight marker (BioRad Precision Plus Protein Dual Color Standard) was loaded to visualize protein size. Blots were probed with antibodies for PSyn, aSyn, MBP, Calbindin and GAPDH followed by species appropriate secondary with horseradish peroxidase and visualized with chemiluminescence. Blots were imaged on a ChemiDoc Imaging System. Blots were stripped and re-probed successively in the following order: P-Syn, T-Syn, MBP and GAPDH, and with a separate blot: MBP, calbindin and GAPDH. Blots were imaged on a ChemiDoc Imaging System. Some residual chemiluminescence can be observed from previous antibodies used.

With little to no change in αSyn and PSyn by IHC, immunoblot or qPCR analysis, we examined whether the αSyn ASO delivered by the 11 amino acid ApoB^11^ peptide was accumulating in affected oligodendrocytes following systemic intra-peritoneal delivery. Biotin labeled αSyn ASO was conjugated to the ApoB^11^ peptide as in earlier experiments and delivered intra-peritoneally to 5-month-old MBP-αSyn tg and non-tg littermate mice. Three days later mice were sacrificed, and brains were analyzed by IHC for the co-localization of biotin labeled αSyn ASO and either Olig2 (oligodendrocytes) or αSyn (Figure 8). Olig2 staining revealed abundant oligodendrocytes in the corpus callosum, cerebellum and brainstem in non-tg mice with fewer numbers in MBP-αSyn tg mice (Figures 8D–F) similar to our IHC observations (Figure 2). Limited biotin labeled αSyn ASO co-localized with the oligodendrocytes despite abundant staining in surrounding areas with only approximately 5% of oligodendrocytes containing ASO (Figures 8A–G) similar to previous published results (Ahammad et al., 2025a,b, p. 1335; (Spencer et al., 2015), p. 1032). Similarly, although the αSyn was observed in the corpus callosum, cerebellum and brainstem of the MBP-αSyn tg mice, there was little co-localization with the biotin labeled αSyn ASO following systemic delivery with less than 5% of αSyn positive cells containing ASO (Figures 8D–F, H). In contrast, biotinylated ASO co-localized at a much greater frequency with both neurons (NeuN) and astrocytes (GFAP) in the corpus callosum, cerebellum and brainstem in both MBP-αSyn tg and non-tg mice (Supplementary Figures S3A–F) Thus, although systemic delivery of αSyn ASO with the ApoB^11^ peptide did cross the BBB and enter the CNS, it reached the target cell (oligodendrocyte) in limited capacity in the MBP-αSyn tg mouse model of MSA-C.

## 4 Discussion

We investigated the systemic delivery of an ASO directed at αSyn for the treatment of MSA neuropathological changes. Treatment of female MBP-αSyn tg mice with ASO reduced gliosis and increased both oligodendrocyte numbers as well as myelination without any measurable impact on accumulation of αSyn. Although ApoB^11^ peptide delivered the αSyn to the CNS, analysis showed limited uptake by oligodendrocytes with uptake instead by surrounding neurons and astrocytes. This suggests a possible bystander effect whereby reducing αSyn in neurons and/or astrocytes has a beneficial effect on oligodendrocytes. It is worth mentioning that the αSyn ASO targets not only the human transgene expressed in oligodendrocytes but also the endogenous mouse αSyn that would be expressed in neurons (Spencer et al., 2019; Leitao et al., 2023a).

Previous use of the 38 amino acid ApoB LDLR binding domain (ApoB^38^) successfully delivered proteins to 20–30% of neurons and to 10–20% of glial cells across the whole brain; however, delivery to oligodendrocytes occurs at a significantly lower rate of ~1–2% (Spencer et al., 2015). However, it was not known how the ApoB^11^ peptide conjugated with an ASO would distribute in the brain. We show here that the ApoB^11^ peptide successfully delivers an ASO to neurons and astrocytes similar to delivery of protein with the ApoB^38^ (Supplementary Figure S3); however, delivery to oligodendrocytes appears to be limited at around 5% (Figure 8). Reports suggest that oligodendrocytes have LDLR and VLDLR expression for uptake of locally synthesized cholesterol to be integrated in myelin production (Zhao et al., 2007; Saher and Stumpf, 2015); however, hypoxic ischemia resulted in reduced expression LDLR in oligodendrocytes in a mouse (Xie et al., 2021). It is not understood why we observe very little uptake of the ApoB^11^ or ApoB^38^ by oligodendrocytes in light of the expression pattern of the LDLR.

Treatment of the MBP-αSyn tg mouse model of MSA-C with ApoB^11^-αSyn ASO did not appear to reduce the accumulation of αSyn or PSyn by IHC or immunoblot. Despite this, we did measure increased myelination, and an increased number of oligodendrocytes coupled with decreased gliosis by IHC. This suggests that the treatment had some effect on the MSA pathology. In view of the limited delivery of ASO to oligodendrocytes, there may have been a small reduction in αSyn accumulation as observed in the cerebellum (Figures 4, 8). In this view, even a small change in αSyn accumulation in oligodendrocytes may be enough to cause a positive outcome in MSA. Western blot and qPCR may not be detecting small changes in myelin basic protein or RNA that are observed by IHC. Protein and RNA analysis were conducted on large portions of the brain that were sub-dissected whereas IHC was measured locally in specific regions. It cannot be ruled out that the αSyn ASO did reduce αSyn expression at a low level in the oligodendrocytes which was observed as increased myelination; however, this was not detected by Western blot or qPCR. Alternatively, studies have shown cell to cell propagation of αSyn by extracellular endocytosis (Desplats et al., 2009), endocytic vesicular transport (Ngolab et al., 2017) and even nanotubes (Dieriks et al., 2017). In fact, we have observed therapeutic reduction in oligodendrocyte αSyn accumulation in another mouse model of MSA following systemic delivery of neurosin, an αSyn degrading enzyme, or an anti-αSyn antibody using the ApoB^38^ peptide delivery method (Spencer et al., 2015; Valera et al., 2017). This suggests that targeting αSyn outside of oligodendrocytes can meaningfully impact αSyn GCI without directly targeting expression of the αSyn RNA transcript as attempted in this study.

While pharmacokinetics were not performed during this study, a more recent study completed by us suggests that systemic administration of ApoB^11^ delivered ASO reaches the brain within 1 h of injection with a half-life of approximately 28 days (Ahammad et al., 2025a,b). This aligns with previous investigation of a ApoB^11^ delivery of an siRNA to a mouse model of PD where we performed repeated administration every 30 days (Spencer et al., 2019; Leitao et al., 2023a). For this study we utilized the MBP29 strain of the MBP-αSyn tg mouse which expressed high levels of αSyn with accumulation in the cortex, corpus callosum and cerebellum. The accelerated accumulation of αSyn in the MBP29 mouse limited out study length due to early death (~6 months) of the transgenic mice. However, longer term studies with a model expressing lower levels of αSyn might elicit more promising results. Indeed, the MBP1 strain of the same mouse line expresses significantly lower levels of αSyn (~20% less) and has a longer expected life span (~18 months) (Shults et al., 2005) and might be better suited to long term delivery and therapeutic benefit described here.

The MBP29 αSyn tg mice show elevated gliosis consisting of increased microglial and astrocyte cells in the gray matter similar to MSA-C patients (Shults et al., 2005; Hoffmann et al., 2019). We also observed increased gliosis (astrocytes) in the brainstem as well as the cortex and the thalamus (Figure 5). Similarly, we observed increased microglia in the cortex, cerebellum and to a lesser extent the brainstem and the thalamus of the MBP29 mice (Figure 6). Treatment with the αSyn ASO reduced the astrocyte cell numbers associated with the MBP29 mice to levels comparable in non-tg mice; however, there was no effect on the microglial numbers.

Previous characterization of the MBP29 strain of MBP-αSyn tg mice showed elevated levels of oligodendrocytes in the corpus callosum and cerebellum accompanied by myelin loss at 3–4 months of age (Ettle et al., 2016; Meszaros et al., 2021). We found decreased numbers of oligodendrocytes in the cortex, cerebellum and brainstem and no change in the corpus callosum at 5 months of age (Figure 2). It is possible that increased GCI in the oligodendrocytes, while in the short term might result in proliferation, in the long term (~5 months of age) leads to loss of cells as a result of toxicity. In fact, increased apoptosis in oligodendrocytes at 9 months has been observed in a slower progressing MBP-αSyn tg mouse line (Line 1) (May et al., 2014). Treatment with the ApoB^11^-αSyn ASO restored the oligodendrocytes or at least prevented their loss at 5 months of age. We confirmed previous findings of myelin loss and reduction in MBP protein (May et al., 2014; Ettle et al., 2016), and treatment with ApoB^11^-αSyn ASO prevented the loss of myelin so that myelination appeared similar to age-matched non-tg littermates.

One limitation of this study was the use of the MBP29 mouse model of MSA. This mouse exhibits significantly greater αSyn expression and increased pathology at an earlier age compared to the MBP1 mouse developed at the same time (Shults et al., 2005). Our previous work on potential therapies for MSA had been primarily examined in the context of the slower MBP1 mouse line so that therapeutics in the MBP29 mouse may be ineffective in a model that so quickly developed pathology associated with αSyn oligodendroglia expression (Ubhi et al., 2010, 2012; Spencer et al., 2015). Another limitation of this study is the use of all female αSyn transgenic mice to model MSA whereas MSA is known to affect males and females in approximately equal proportion (Reddy and Dieriks, 2022). Previous studies had utilized only female MBP29 mice, so we followed this approach in order to evaluate known deficits in the transgenic mouse (Ubhi et al., 2010; Spencer et al., 2015; Ettle et al., 2016).

## 5 Conclusions

Systemic delivery of αSyn ASO through BBB transport with the peptide ApoB^11^ increased myelination in the corpus callosum and the cerebellum and increased numbers of oligodendrocytes while reducing gliosis in MBP-αSyn tg mice. However, lack of significant delivery of the αSyn ASO to oligodendrocytes may have tempered the overall effect of this therapeutic approach as no change in overall αSyn or PSyn was detected in any of the brain regions examined. Thus, the use of the ApoB^11^ peptide for delivery of ASO to oligodendrocytes for therapeutic approaches may need to be utilized in conjunction with other therapeutic approaches that more directly reduce the accumulation of αSyn.

## Data Availability

The raw data supporting the conclusions of this article will be made available by the authors, without undue reservation.

## Supplementary material

Supplementary Figure S1 Diagram of experimental plan. MBP-αSyn tg and non-tg female mice 3-months of age were treated with intra-peritoneal injections of ApoB^11^-αSyn or SCR ASO at 2 mg/kg twice 3 weeks apart. Two months after the first injection, mice were sacrificed and analyzed by immunohistochemistry, Western blot and qPCR.

Supplementary Figure S2 Expression of human αSyn and MBP RNA in MBP-αSyn tg mice. cDNA generated from cortex, cerebellum and brainstem RNA was analyzed by real-time PCR with primer/probes specific for **(A)** human αSyn and **(B)** mouse MBP from MBP-αSyn tg and non-tg mice treated with ApoB^11^-Scr ASO or αSyn ASO and normalized to non-tg treated with ApoB^11^-Scr ASO expression levels. Data represent mean ± SEM, *N* = 4 mice per group.

Supplementary Figure S3 Co-localization of biotinylated αSyn ASO with neurons (NeuN) and astrocytes (GFAP). Representative regions double labeled for αSyn ASO (red) and neurons (NeuN, green) or astrocytes (GFAP, green) in **(A–C)** non-tg and **(D–F)** MBP-αSyn tg mice treated with ApoB^11^-αSyn ASO (biotinylated) in **(A, D)** cortex or corpus callosum, **(B, E)** cerebellum, and **(C, F)** brainstem imaged with a LSCM. Scale bars = 50 μm. *N* = 3 mice per group.

Supplementary Figure S4 Native gel analysis of αSyn in MBP-αSyn tg and non-tg mice treated with ApoB^11^-Scr ASO or αSyn ASO. Native gels were run with non-reduced protein samples and blotted for analysis. **(A)** Representative immunoblots from cortex, cerebellum, and brainstem enriched regions analyzed with antibodies specific for αSyn and ß-actin. Graphs represent quantitation of immunoblots showing relative levels of **(B)** αSyn protein normalized to ß-actin and then normalized to wild type treated with ApoB^11^-Scr ASO. Data represent mean ± SEM.
