# Supplementary figures and images for: Systemic delivery of anti-sense oligonucleotide targeting α-synuclein for treatment in a mouse model of multiple system atrophy
Source: Front Aging Neurosci. 2026 Apr 29;18:1807721. doi: 10.3389/fnagi.2026.1807721 (PMC13168115; doi:10.3389/fnagi.2026.1807721)

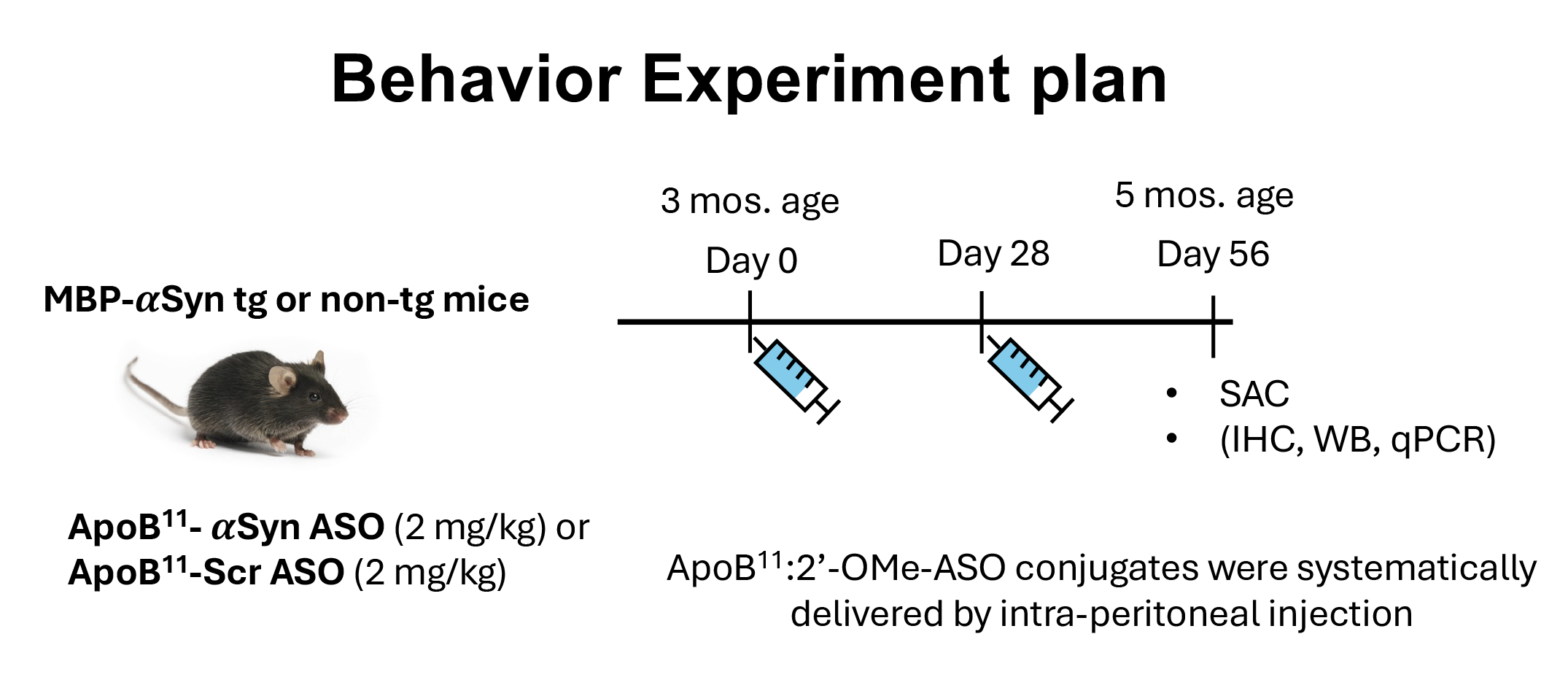

Supplement: Supplementary Figure S1 — Diagram of experimental plan. MBP-αSyn tg and non-tg female mice 3-months of age were treated with intra-peritoneal injections of ApoB11-αSyn or SCR ASO at 2 mg/kg twice 3 weeks apart. Two months after the first injection, mice were sacrificed and analyzed by immunohistochemistry, Western blot and qPCR. [file Figure_1.tif]

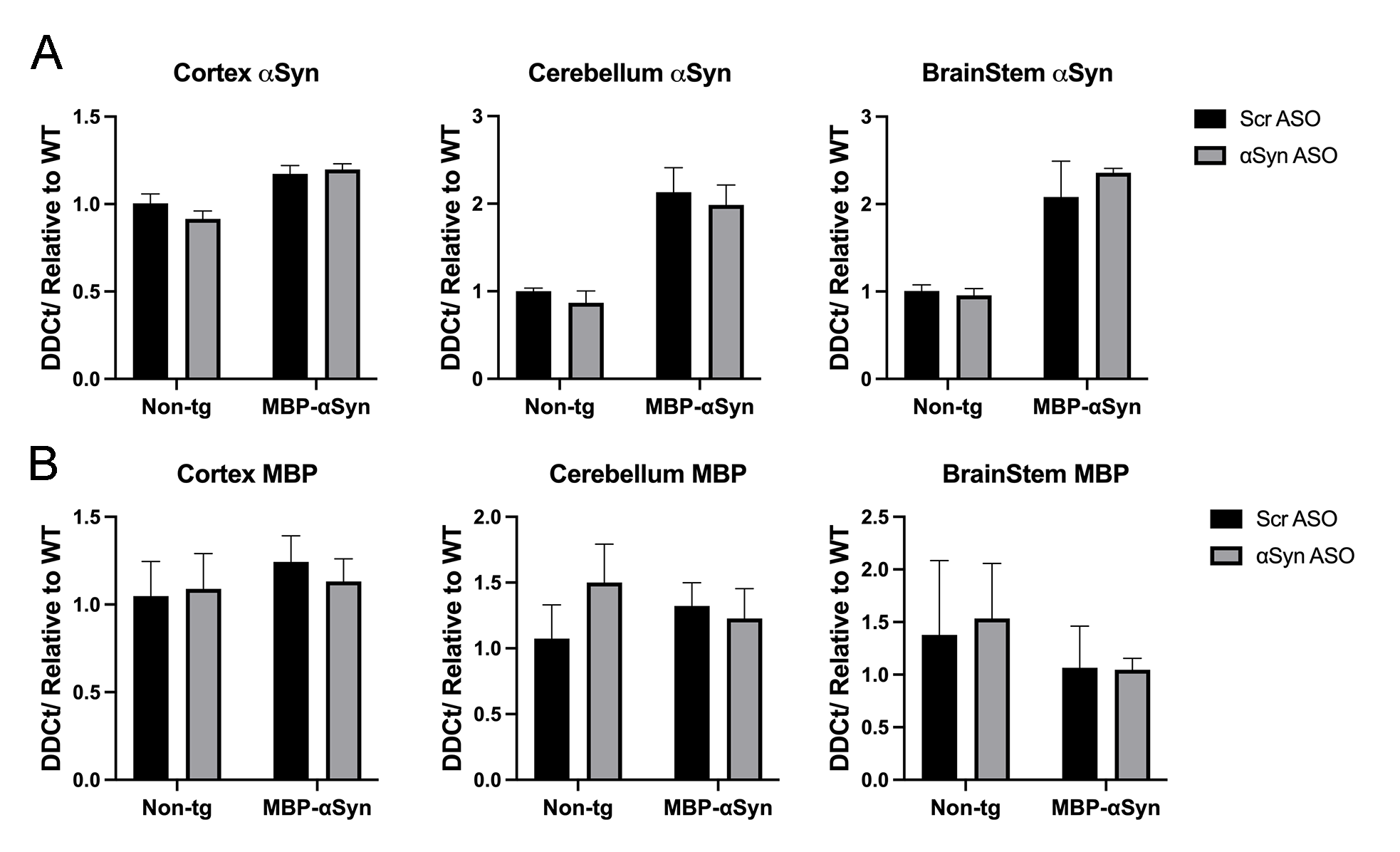

Supplement: Supplementary Figure S2 — Expression of human αSyn and MBP RNA in MBP-αSyn tg mice. cDNA generated from cortex, cerebellum and brainstem RNA was analyzed by real-time PCR with primer/probes specific for (A) human αSyn and (B) mouse MBP from MBP-αSyn tg and non-tg mice treated with ApoB11-Scr ASO or αSyn ASO and normalized to non-tg treated with ApoB11-Scr ASO expression levels. Data represent mean ± SEM, N = 4 mice per group. [file Figure_2.tif]

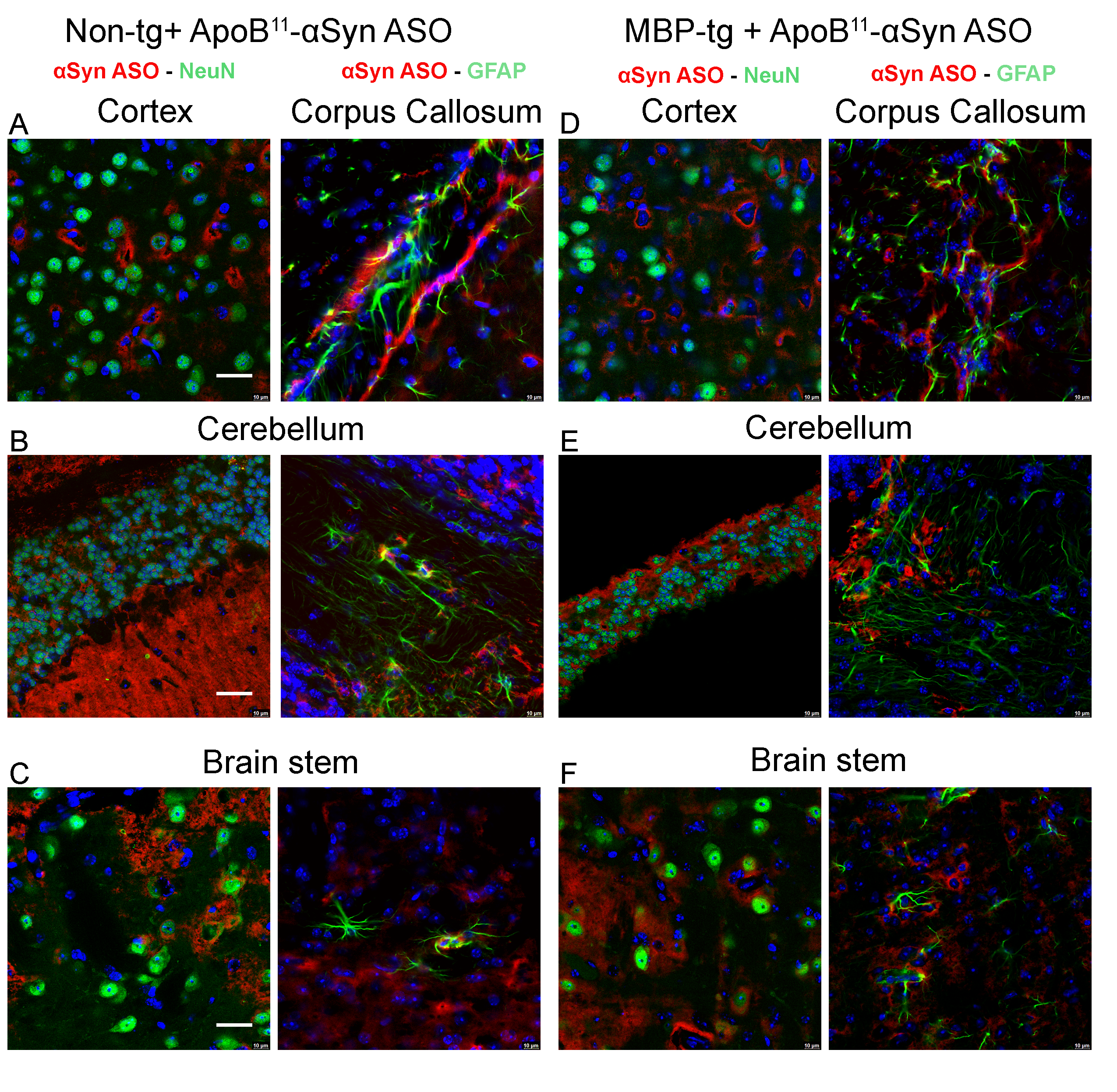

Supplement: Supplementary Figure S3 — Co-localization of biotinylated αSyn ASO with neurons (NeuN) and astrocytes (GFAP). Representative regions double labeled for αSyn ASO (red) and neurons (NeuN, green) or astrocytes (GFAP, green) in (A–C) non-tg and (D–F) MBP-αSyn tg mice treated with ApoB11-αSyn ASO (biotinylated) in (A, D) cortex or corpus callosum, (B, E) cerebellum, and (C, F) brainstem imaged with a LSCM. Scale bars = 50 μm. N = 3 mice per group. [file Figure_3.tif]

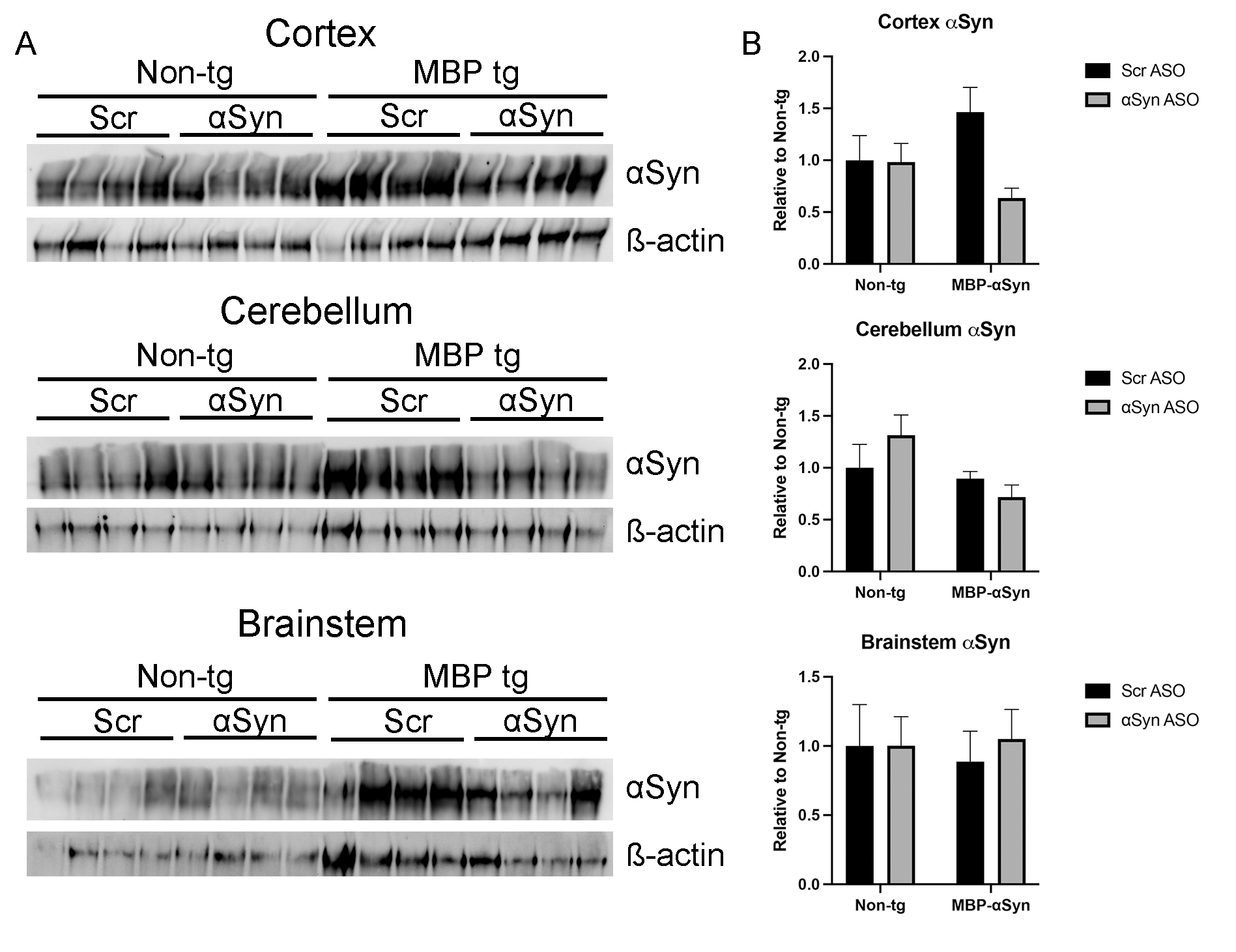

Supplement: Supplementary Figure S4 — Native gel analysis of αSyn in MBP-αSyn tg and non-tg mice treated with ApoB11-Scr ASO or αSyn ASO. Native gels were run with non-reduced protein samples and blotted for analysis. (A) Representative immunoblots from cortex, cerebellum, and brainstem enriched regions analyzed with antibodies specific for αSyn and ß-actin. Graphs represent quantitation of immunoblots showing relative levels of (B) αSyn protein normalized to ß-actin and then normalized to wild type treated with ApoB11-Scr ASO. Data represent mean ± SEM. [file Figure_4.tif]
